# The Predictive Role of the Posterior Cerebellum in the Processing of Dynamic Emotions

**DOI:** 10.1007/s12311-023-01574-w

**Published:** 2023-06-07

**Authors:** Gianluca Malatesta, Anita D’Anselmo, Giulia Prete, Chiara Lucafò, Letizia Faieta, Luca Tommasi

**Affiliations:** Department of Psychological, Health and Territorial Sciences - University “G. d’Annunzio” of Chieti-Pescara, Chieti, Italy

**Keywords:** Neuromodulation, Neuropsychology, Facial expressions, Emotion, Prediction

## Abstract

Recent studies have bolstered the important role of the cerebellum in high-level socio-affective functions. In particular, neuroscientific evidence shows that the posterior cerebellum is involved in social cognition and emotion processing, presumably through its involvement in temporal processing and in predicting the outcomes of social sequences. We used cerebellar transcranial random noise stimulation (ctRNS) targeting the posterior cerebellum to affect the performance of 32 healthy participants during an emotion discrimination task, including both static and dynamic facial expressions (i.e., transitioning from a static neutral image to a happy/sad emotion). ctRNS, compared to the sham condition, significantly reduced the participants’ accuracy to discriminate static sad facial expressions, but it increased participants’ accuracy to discriminate dynamic sad facial expressions. No effects emerged with happy faces. These findings may suggest the existence of two different circuits in the posterior cerebellum for the processing of negative emotional stimuli: a first-time-independent mechanism which can be selectively disrupted by ctRNS, and a second time-dependent mechanism of predictive "sequence detection" which can be selectively enhanced by ctRNS. This latter mechanism might be included among the cerebellar operational models constantly engaged in the rapid adjustment of social predictions based on dynamic behavioral information inherent to others’ actions. We speculate that it might be one of the basic principles underlying the understanding of other individuals’ social and emotional behaviors during interactions.

## Introduction

Facial emotional expressions are a primary form of nonverbal communication and play a critical role in human interactions [1], since the ability to accurately perceive and interpret them is essential for successful social communication [2, 3]. While the role of the cerebral cortex in facial emotion perception has been extensively investigated [1, 4–10], the contribution of the cerebellum to this process is comparatively less understood. However, recent lesion, neuroimaging, and neuromodulation studies have increasingly provided compelling evidence that the cerebellum—for more than a century thought to be exclusively involved in motor coordination and learning—is actually involved in high-level cognitive functions [11–15] including social cognition [11, 16–32] and emotion processing [33–51]. More specifically, a difference has been proposed between the anterior lobe of the cerebellum, more engaged in sensorimotor control, and the posterior lobe, more engaged in cognitive, social, and emotional functions [12, 14, 30, 31, 37, 44, 52]. Concerning the role of the cerebellum in emotion processing, most of the studies carried out so far have exploited emotion discrimination and recognition tasks in which static images (photographs) depicting facial emotional expressions are presented to patients/participants [16, 52–68]. Based on these findings, it is therefore conceivable that the cerebellum is involved in the perception of facial emotional expressions, especially in response to negative emotions (i.e., sadness, anger, and fear) and aversive stimuli [16, 36, 37, 41, 46, 52–54, 56, 58, 59, 62, 65, 67, 69–76]. In fact, neuroimaging evidence showed emotion-specific activations in the cerebellum (along with the posterior cingulate and fusiform gyrus) in response to negative, but not positive, facial emotions [67]. According to this view, posterior areas of the cerebellum would rapidly kick off a set of ancestral defense mechanisms, such as fight-or-flight motor reactions (in response to potentially dangerous stimuli coming from outside) which in turn involve more anterior cerebellar areas [34, 70, 73, 77, 78].

Although several operating mechanisms of the cerebellum are still not fully understood, it seems plausible to state that both the motor and the non-motor (i.e., cognitive/social/emotional) cerebellum work through operating principles that are analogous [13, 15, 79]. The recent view is that the cerebellum is continuously involved in two adaptive and interdependent functions: prediction and anticipation. More specifically, cerebellar operational models and circuits would detect and anticipate the presence of temporal and spatial patterns from physical cues, make accurate predictions of prospective outcomes on the basis of these cues, and prepare to respond accordingly [15, 23, 80–86]. Within this framework, it has been suggested that the operational mode of the cerebellum in prediction and anticipation processes—even beyond the motor domain—is a time-related “sequence detection” [15, 80, 82, 87–91]. The cerebellar automatic mechanism of prediction (i.e., generating expectations) and anticipation (i.e., preparing for future events) of sequences, as well as the rapid adjustment of such predictions based on dynamic environmental information, would be one of the leading processes underlying the understanding of other individuals’ behaviors and mental states during social interactions [18, 22, 23, 25, 88, 92–94]. Nevertheless, to date, no human study has explored the causal role of the cerebellum in this putative prediction/anticipation mechanism within the specific domain of facial emotion processing. One way to investigate this process in the field of emotions is by exploiting the presentation of dynamic emotional facial expressions, which have been gradually recognized as a more sensitive and ecologically valid stimulus compared to the static presentation of emotional faces [95]. In this regard, dynamic negative emotions, as opposed to positive emotions, have been shown to elicit activation patterns in the cerebellum (along with the left inferior frontal gyrus and the pars orbitalis [66]).

In recent years, the use of non-invasive brain stimulation techniques (e.g., transcranial magnetic stimulation and transcranial electrical stimulation) for studying cerebellar functions in non-motor domains (such as cognition, social behavior, and emotion) has substantially increased [25, 59, 60, 65, 75, 92, 96–105]. These techniques can help neuroscientists to provide causal links between cerebellar activity and behavior. In this study, we exploited cerebellar transcranial random-noise stimulation (ctRNS; i.e., a non-invasive technique of electrical brain stimulation, the usefulness of which has already been proven in several cognitive and emotional processes in the neocortex [106–111]) in order to causally verify the role of the cerebellum in predicting and anticipating sequences of facial emotional expressions changing over time. This specific stimulation technique can be applied either at low or high frequency with different effects on the target area of the brain, and cortical stimulation research showed that high-frequency tRNS led to excitatory effects [112]. Here, we used an emotion discrimination task in which participants were required to quickly respond to videos of faces that progressively evolved from a neutral to either a negative (sad) or a positive (happy) emotional expression [113]. Based on the above-mentioned evidence that the cerebellum would play a role in the processing of emotional expressions, especially for negative valence, and that its operational mechanism would include the ability to understand the timing and predict the outcomes of physical and social action sequences, we hypothesized that high-frequency ctRNS over the posterior cerebellum could enhance participants’ performance in a discrimination task of dynamic—especially negative—facial expressions of emotion.

## Materials and Methods

### Participants

Thirty-two participants took part in the study (16 females, mean age ± standard deviation: 24.56 ± 3.5 years). Handedness was assessed by using the Edinburgh Handedness Inventory [114, 115], according to which the handedness score ranges from − 100 (totally left-handed) to + 100 (totally right-handed): the mean handedness score was 46.97 (± 42.26) including four left-handed. All participants were free from any history of psychiatric or neurological disorders and gave written consent before taking part to the experiment. The whole procedure was carried out in accordance with the principles of the Declaration of Helsinki and was approved by the Institutional Review Board of Psychology of the Department of Psychological, Health and Territorial Sciences—University “G. d’Annunzio” of Chieti-Pescara (protocol number: IRBP/22002).

### ctRNS and General Procedure

High-frequency ctRNS was delivered by a battery-driven, constant current stimulator (DC-Stimulator, NeuroConn GmbH, Germany) through a pair of surface saline-soaked sponge electrodes, one measuring 5 cm × 5 cm and the other measuring 5 cm × 10 cm, kept firm by elastic bands. A random noise current was applied for 20 min with an intensity of 1.5 mA and with a 0 mA offset. Random frequencies ranged from 100 to 640 Hz (high frequency), according to safety guidelines, with a ramping period of 15 s both at the beginning and at the end of the stimulation.

**Fig. 1:**
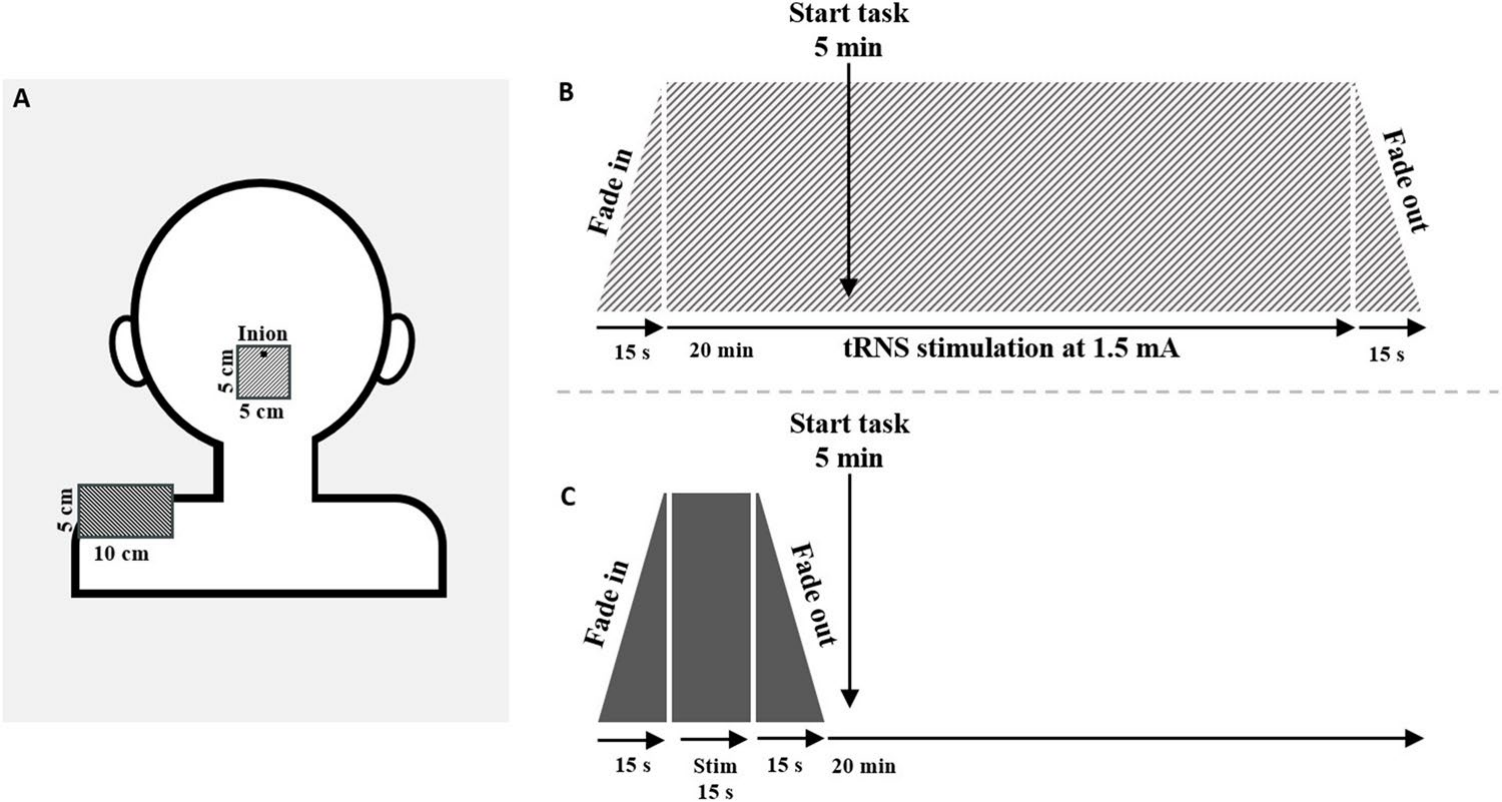
**A** Schematic representation of electrodes placement (smaller electrode centered on the cerebellar cortex with the center placed 2 cm below the inion, and a larger electrode placed on the left shoulder); **B** timeline of real ctRNS condition; **C** timeline of sham condition

All participants took part in two different stimulation sessions (ctRNS and sham), carried out on 2 different days separated by at least 24 h. The smallest electrode was centered on the cerebellar cortex (the center of the electrode was placed 2 cm below the inion [25]), and the largest electrode was placed on the left or right shoulder (balanced between participants). In the sham condition, the current was turned off after 15 s, and the order of the two sessions was counterbalanced across participants. The task started 5 min after the beginning of the stimulation and was carried out online (see Fig. 1). The experiment was run using E-Prime 3.0 software (Psychology Software Tools, Inc., Pittsburgh, PA) on a Windows personal computer. Participants were required to comfortably sit in a quiet room, at about 57 cm from the computer screen.

At the end of each session, participants were required to complete a questionnaire investigating their sensation concerning stimulation [116]. None of the participants reported feeling strong pain or burning sensations, a minority of them (16%) reported feeling mild pain and burning sensations in both sessions.

### Stimuli and Procedure

**Fig. 2:**
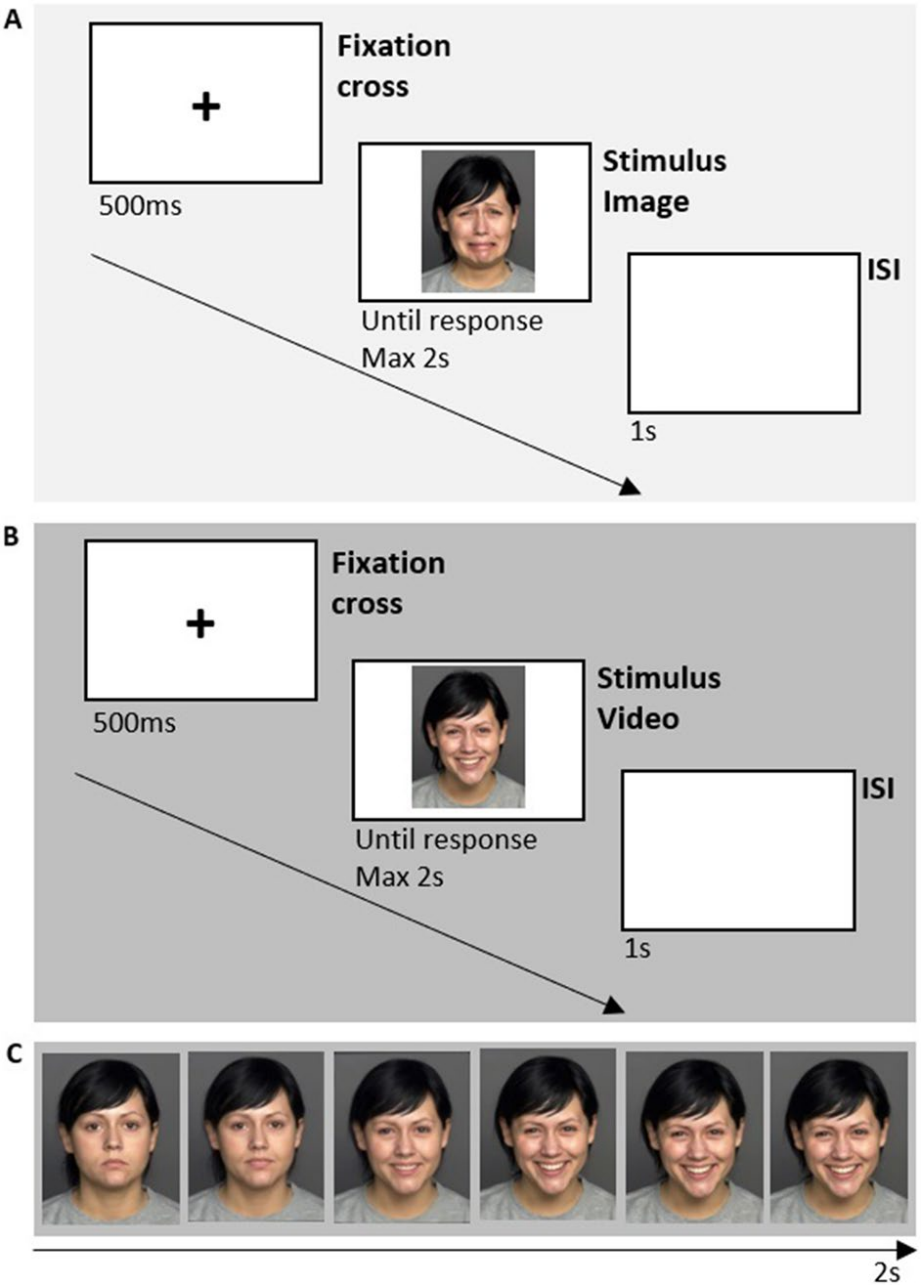
Timeline of experimental trials for **A** static (sad) condition and **B** dynamic (happy) condition; **C** sample of a video stimulus showing morph images captured every 400 ms from the start of the video (from neutral to happy expression). The photographs/video depict the person corresponding to the n. 140, taken respectively from the FACES and Dynamic FACES databases (permitted for publication according to the FACES Platform Release Agreement)

The task comprised a static and a dynamic condition. The static condition used color photographs of face images (resolution of 2.835 × 3.543 pixels) from the FACES database [117]. Photos of 12 males and 12 females displaying happy and sad facial expressions were selected. The images were also flipped horizontally, so the final set of stimuli comprised 96 static faces. In the dynamic condition, stimuli consisted of video clips (frame rate of 30 fps and resolution of 384 × 480 pixels) obtained by morphing faces selected from the Dynamic FACES database [113]. The videos started with the presentation of a neutral face that progressively evolved into a happy or sad expression (see Fig. 2 C). Each video had a duration of two seconds: morphing took place in the first second and was followed by one second in which the expression was static. Videos from the same actors of the static session (12 males and 12 females) were selected. The videos were also flipped horizontally, so the final set of stimuli comprised 96 video clips.

In each stimulation session (ctRNS and sham), participants performed both the static and the dynamic condition, in a counterbalanced order. In each condition, 96 trials were presented (either images or video alternatively). In each trial, a black fixation cross was presented for 500 ms, and it was followed by either an image or a video presented in the center of the screen for a maximum of 2 s or until participants gave the response. Then, after the presentation of an interstimulus interval (ISI) lasting 1 s, the next trial started. Participants were asked to classify each stimulus as happy or sad, stopping the presentation of the image or interrupting the morphing video as soon as they recognized the emotion displayed (see Fig. 2 A and B). Responses were provided by pressing two different keys with the right hand, corresponding to the happy and the sad expression, respectively (the association between key and response was balanced across participants).

## Results

Data analysis was based on the proportion of correct responses (accuracy) in categorizing the emotional expressions (calculated by dividing the number of correct responses by the total number of stimuli and multiplying by 100%) and on the response times (RTs) in milliseconds for correct responses (calculated from the begin of the presentation of the static or dynamic stimulus). Responses with RTs values deviating by more than two standard deviations from the subject’s mean for any condition (static and dynamic) were excluded. Accuracy and RTs were used as the dependent variables in two repeated measures analyses of variance (ANOVA) with the stimulus (static, dynamic), emotion (happy, sad), and session (ctRNS, sham) as within-subjects factors. All statistical analyses were computed by means of Statistica 8.0 software (StatSoft. Inc., Tulsa, USA) and when needed, the Duncan test was used for post-hoc comparisons (*p* < 0.05).

**Fig. 3:**
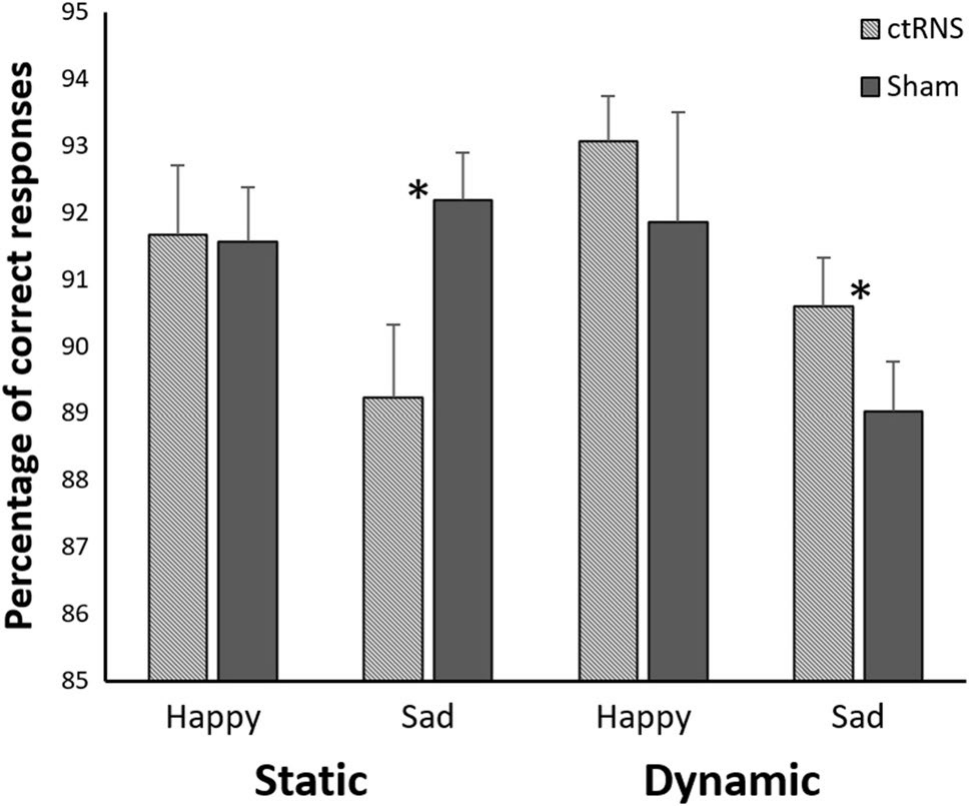
Interaction among stimulus (static, dynamic), emotion (happy, sad), and session (ctRNS, sham) on the accuracy (percentage of correct responses) in the categorization of emotional expression. Bars represent standard errors and asterisks show significant differences

As regards accuracy, the main effect of emotion was significant (*F*_1,31_ = 7.61, *p* = 0.009, ηp2 = 0.20), showing a higher accuracy for happy (92.04 ± 0.79) than for sad faces (90.26 ± 0.51). The interaction among stimulus, emotion, and session was significant (*F*_1,31_ = 6.07, *p* = 0.022, ηp2 = 0.16; Fig. 3), and post hoc comparisons revealed that in the static condition, sad expression was categorized worse during ctRNS than during sham (*p* < 0.001); however, in the dynamic condition sad expression was categorized better during ctRNS than during sham (*p* = 0.045). No difference was observed for happy expression in both static and dynamic conditions.

As regards RTs, the main effect of the stimulus was significant (*F*_1,31_ = 129.50, *p* < 0.001, ηp2 = 0.81), with higher RTs for dynamic (725.96 ± 21.91 ms) than for static stimuli (559.89 ± 21.89 ms). The main effect of emotion was also significant (*F*_1,31_ = 61.59, *p* < 0.001, ηp2 = 0.67), with higher RTs for sad (658.51 ± 21.36 ms) than for happy expression (627.34 ± 20.11 ms). The interaction between stimulus and emotion (*F*_1,31_ = 12.26, *p* = 0.001, ηp2 = 0.28) showed that the happy expression was categorized faster than the sad expression, in both static (*p* < 0.001) and dynamic conditions (*p* < 0.001).

## Discussion

In this study, we investigated the causal role of the posterior cerebellum in categorizing positive (happy) and negative (sad) facial emotions, presented as static and—importantly—as dynamic stimuli. This is the first neuromodulation study making use of this type of stimuli in cerebellar research, although a number of neuroimaging studies have included the cerebellum among the structures involved in the neural pathways for the perception of dynamic facial emotions [52, 95, 118–121]. In addition, for the sake of comparison with previous cerebellar studies on emotion processing, we still used a set of static emotional images of facial expressions. Our hypothesis was that high-frequency ctRNS over the posterior cerebellum would enhance participants’ performance in emotion discrimination, especially in response to dynamic negative (i.e., sad) facial expressions.

First of all, results unsurprisingly showed that participants performed better (in terms of higher accuracy and lower RTs) for happy than for sad facial expressions. In this regard, it has been suggested that the teeth exposure during a happy smile produces a circumscribed contrasting luminance in the mouth area capable of capturing the observer’s attention and thus facilitating the detection of happiness as shown both through discrimination and visual search tasks [122–124].

More interestingly, results showed a triple significant interaction revealing an inverse pattern of participants’ performance accuracy in response to negative static vs. dynamic stimuli during ctRNS. In fact, while on the one hand ctRNS compared to the sham condition significantly *reduced* participants’ accuracy to discriminate *static* sad facial expressions, on the other hand, ctRNS significantly *increased* participants’ accuracy to discriminate *dynamic* sad facial expressions. As no difference was found in response to the positive (i.e., happy) stimuli, our findings primarily confirm the idea that the posterior cerebellum is selectively involved in a circuit for the discrimination of the static facial cues deriving from negative emotional expressions [16, 36, 37, 41, 46, 52–54, 56, 58, 59, 62, 65, 67, 69–76] and that this circuit can be altered by high-frequency ctRNS applied over the posterior cerebellum. As suggested by previous studies, this might be due to the greater biological and evolutionary salience of negative emotions, along with the crucial role of the cerebellum in the consequent preparation of a rapid motor response [34, 70, 73, 77, 78]. However, since our results showed that ctRNS selectively and oppositely altered participants’ performance accuracy for static—with a decrease—and dynamic—with an increase—sad facial expressions, we speculate that two different circuits exist for the processing of negative emotional stimuli in the cerebellum: a first time-independent mechanism which can be selectively disrupted by ctRNS, and a second time-dependent mechanism of predictive “sequence detection” which can be selectively enhanced by ctRNS. It has to be noted that our speculations must be considered with caution due to some possible limitations of the study. First, based on previous studies (e.g., [25]), we used the inion as a reference for the placement of the cephalic electrode, but only the use of neuronavigation could ensure that the same target area is stimulated in all participants. In this regard, it can be pointed out that the size of the electrodes used in the tES protocol is quite large to avoid the need for a high spatial resolution, but this issue can be at the basis of inter-individual differences in the targeted cerebellar area. However, the electrode montage we used on the posterior cerebellum provides us with a reasonable certainty that we modulated cerebellar activity in the posterior vermal and paravermal areas, as well as the adjacent regions of the posterior cerebellar hemispheres. Second, the literature about ctRNS is not so wide to conclusively support either the facilitatory or inhibitory effects of this stimulation set-up on the cerebellar cortex. Nevertheless, the use of random noise stimulation should ensure that stochastic resonance effects occur, so that we can be confident about the overall excitatory induction of the targeted area [125]. Even in this regard, however, neuroimaging studies investigating the direct effect of hf-tRNS applied on the cerebellum are needed to directly corroborate its effect. Furthermore, even if, at a pure statistical level, the three-way interaction revealed a participants’ lower accuracy in the discrimination of static sad expressions and a higher accuracy in the discrimination of dynamic sad expressions (with respect to the corresponding sham conditions), the mean accuracies of the sample show that this latter difference seems to be attributable to a decreased accuracy for the dynamic sad stimuli in sham condition, more than to an enhancement of the performance during the active stimulation. This means that if we consider only the sham sessions, the participants’ accuracy is lowest in the dynamic-sad condition than in all the other static and dynamic conditions. This difference among the sham sessions can suggest that sadness is not so simple to detect as the happiness expression. As specified above, in fact, this is in line with the evidence of a facilitation in the detection of smiling faces, possibly due to teeth exposure [122–124]. Importantly, the contrasting patterns obtained for static vs. dynamic sad expressions are in line with the specific role of ctRNS in sadness perception. Finally, it should be noted that dynamic emotions should have increased participants’ perceived realism of facial expressions, because dynamic compared to static stimuli facilitate the processing of complex and changing cues that constantly occur during real social interactions [95, 126–129].

## Conclusion

This study showed that a cerebellar automatic mechanism of predicting and anticipating temporal sequences exists not only for the prediction of social action sequences, but also for predicting the behavioral outcome of *emotional* sequences (i.e., dynamic facial expressions). Since this ability can be differentially altered by ctRNS applied over the posterior cerebellum for static and dynamic stimuli, we can conclude that it could be a different mechanism compared to that investigated so far by most of the studies carried out in the emotional domain [16, 36, 37, 41, 46, 52–54, 56, 58, 59, 62, 65, 67, 69–76]. In fact, it is possible that this mechanism is part of the already-known cerebellar operational models constantly engaged in the rapid adjustment of social predictions based on dynamic environmental information, which are presumably at the root of the understanding of other individuals’ social and emotional behaviors during interactions [18, 22, 23, 25, 92–94]. In our opinion, these findings support the recent idea of the cerebellum as the central coordinator of the structures involved in the “predictive brain” [80, 91]. Finally, the present study showed that ctRNS is a useful technique for altering the activity of the cerebellum, although further studies are needed to better understand the dimensions and directionality of such alterations, as well as the existence of such a putative mechanism of predictive “sequence detection,” specifically involved in the emotional processing of negative facial expressions.

## Data Availability

Data and materials of this study are available from the corresponding author, G.M., upon reasonable request.
